# 

**Published:** 1878-09

**Authors:** 


### CONTENTS:

ARTICLE I.—The American Dental Association........ 193

ARTICLE II.—Relations of the Dental Pulp to other Tooth Tissues. By L. C. Ingersoll........ 210

ARTICLE III.—Conservative Treatment of the Pulp. By Walter H. Fundenberg, D. D. S........ 216

ARTICLE IV.—The Metric System in a Nut-Shell. By Edward Wigglesworth, M. D. 223

ARTICLE V.—The Southern Dental Association........ 227

ARTICLE VI.—Pyorrhœa Alveolaris.—A Case in Practice. By W. H. Eames, D. D. S........ 230

### EDITORIAL, ETC.

Editorial Correspondence........ 233

Mal-Nutrition,—Its Causes........ 235

### BIBLIOGRAPHICAL.

Questions on the Structure and Development of Human Teeth........ 236

### MONTHLY SUMMARY.

Impurities and Tests of Chloroform........ 237
Treatment of Furruncles by Arnica........ 238
Therapeutic Value of Nitrite of Lead........ 238
Stilbeine for Cleaning Instruments........ 238
Skin Grafting in the Colored Races........ 239
Death under Ether........ 239
The Antiseptic and Therapeutic Properties of Boracic Acid........ 240
Vermilion........ 240

